# Pets

**Published:** 1877-04

**Authors:** 


### PETS.

Almost every one has a fondness for pets, and the diversity of taste as manifested in the selection of them, is noteworthy. Parrots, monkeys, canarys, cats and dogs compose a list from which a selection is made. It argues well for the household that keeps a pet—it is an indication of a loveable nature, a warm heart, capable of generous impulses. Were we a young man, upon the lookout for a wife, we should give preference to the girl who kept and fondly cared for a pet. The chances are much enhanced from such an inclination that she will make a loveable and loving wife. We greatly distrust any individual who will wantonly abuse a pet. The man that will kick a dog, abuse a cat, or whip a horse, may be set down as a cruel man to wife and children, if he posseses them.

If you would employ a woman to care for your children—to act in the capacity of nurse and teacher to them, ascertain first, with reference to her character, then as to her fondness for pets. If she keeps a bird or cat, it is a good indication, and we would be willing to guarantee her capabilities for the position.

We once had a man apply to us for a position as groom to our horses. He was accompanied by a coach dog that walked demurely behind him. While listening to the recital of his own capabilities for the position, he accidently stepped upon the dog's foot, and then brutally kicked the animal for getting in his way. We promptly answered him that he would not suit us, and the wisdom of our decision became manifest the next day, when his former employer informed us that he discharged him for beating one of his horses. The man was cruel, and revealed his nature in his every act. He had no fondness for pets, but abused then when they came accidently in his way.

Did you ever observe the resemblance of features between a good master and his dog? Not that the master degenerates to an expression of brutality, but that his dog becomes more humanized, so that the expression of the master is revealed in him. This circumstance is noted by Darwin, and we have frequently heard it spoken of and commented upon by those who have observed this phenomenon. It argues well for the master, that he was so beloved by his pet as to grow into a resemblance of him.

Secure to your household a pet, and if we may be allowed to select one for you, and you are entitled to have such a one—let it be a baby, and if it be possible to elect the sex, by all means let it be a girl-baby. We have had all sorts and kinds of pets, you would scarcely believe us did we enumerate them, but the most glorious and satisfactory of them all, is the girl-baby; the house that contains one is blessed beyond compare. Take unto yourselves pets, and if you have a wife to care for it, let it be a baby—a girl-baby.

